# Cases of Amputation in Private Practice, from June, 1833, to January, 1846

**Published:** 1846-02

**Authors:** Thomas F. Betton

**Affiliations:** Germantown, Pa.


*Germantown*, January 1, 1846.

Dr. R. M. Huston:

*Dear Sir:*—If the enclosed is worthy of a place in the Examiner, as showing the results of country air in opposition to that of cities and hospitals, I shall be obliged to you if you will give it some obscure corner.

Very faithfully yours,

Thos. F. Betton.

*Cases of Amputation in Private Practice, from June*, 1833, *to January*, 1846. By Thomas F. Betton, M. D., of Germantown, Pa.

| No | Name. | Age | Sex. | Cause of Removal. | Where Removed. | Result. |
|---|---|---|---|---|---|---|
| 1 | F. G. | 22 | Male. | Fungus Hæmatodes. | Thigh. | Recovered. |
| 2 | W. H. | 35 | Male. | Compound fracture, caused by machinery | Forearm. | Recovered. |
| 3 | A. W. | 12 | Male Black | Fungus Hæmatodes. | Thigh. | Recovered. |
| 4 | H. S. | 50 | Male. | Scrofulous disease of Ankle-joint. | Leg. | Recovered. |
| 5 | M. B. | 13 | Female. | Lacerated by a machine for tearing flax. | Arm. | Recovered. |
| 6 | E. S. | 32 | Female. | Scrofulous disease of Ankle-joint. | Leg. | Recovered. |
| 7 | W. B. | 50 | Male. | Gangrene. | Thigh. | Died. |
| 8 | A. W. | 18 | Female. | Necrosis. | Leg. | Recovered. |
| 9 | R. L. | 12 | Male. | Gunshot wound of Hand. | Forearm. | Recovered. |
| 10 | N. B. | 33 | Male. | Compound fracture from Rocks in a Quarry. | Forearm. | Recovered. |
| 11 | J. H. | 35 | Male. | Compound fracture from premature explosion of a blast. | Forearm. | Recovered. |
| 12 | J. K. | 42 | Female. | Scrofulous disease of leg of many years. | Thigh. | Recovered. |
| 13 | P. L. | 30 | Male. | Compound fracture by a Rock of ½ ton weight falling on the foot. | Leg. | Recovered. |
| 14 | J. C. | 18 | Male. | Hand torn in a cotton carding machine. | Forearm. | Recovered. |
| 15 | B. M. | 30 | Male. | Mashed by a Locomotive Engine. | Both Legs. | Recovered. |

REMARKS.

The only case possessing much interest is No. 15, in which both legs were removed. The man had been thrown down on the railroad by the locomotive, which passed over his legs diagonally, tearing them most horribly, and splintering the bones nearly to the knee-joint. He received, in addition, a severe concussion of the brain; and at the time of the operation was totally unconscious of everything around him—nor did he, for a week subsequently, know that his limbs had been injured. He was, fortunately, a man of strictly temperate habits, which, added to a good constitution, no doubt secured his recovery. A recovery from amputation of both legs, for a recent accident, especially so severe as that produced by a locomotive, is, I believe, uncommon.

